# Habitat modifies the relationship between grass and herbivore species richness in a South African savanna

**DOI:** 10.1002/ece3.11167

**Published:** 2024-04-15

**Authors:** Jan Čuda, Klára Pyšková, Martin Hejda, Llewellyn C. Foxcroft, Sandra MacFadyen, David Storch, Robert Tropek, Guin Zambatis, Petr Pyšek

**Affiliations:** ^1^ Department of Invasion Ecology Czech Academy of Sciences, Institute of Botany Průhonice Czech Republic; ^2^ Department of Ecology, Faculty of Science Charles University Prague Czech Republic; ^3^ Scientific Services, South African National Parks Skukuza South Africa; ^4^ Centre for Invasion Biology, Department of Botany and Zoology Stellenbosch University Matieland South Africa; ^5^ Department of Mathematical Sciences Stellenbosch University Matieland South Africa; ^6^ Centre for Theoretical Studies Charles University Prague Czech Republic; ^7^ Czech Academy of Sciences Biology Centre, Institute of Entomology České Budějovice Czech Republic

**Keywords:** dominance control, grass diversity, grassland, grazer, plant–herbivore interactions, water and nutrient availability

## Abstract

The savanna ecosystem is dominated by grasses, which are a key food source for many species of grazing animals. This relationship creates a diverse mosaic of habitats and contributes to the high grass species richness of savannas. However, how grazing interacts with environmental conditions in determining grass species richness and abundance in savannas is still insufficiently understood. In the Kruger National Park, South Africa, we recorded grass species and estimated their covers in 60 plots 50 × 50 m in size, accounting for varying proximity to water and different bedrocks. To achieve this, we located plots (i) near perennial rivers, near seasonal rivers, and on crests that are distant from all water sources and (ii) on nutrient‐rich basaltic and nutrient‐poor granitic bedrock. The presence and abundance of large herbivores were recorded by 60 camera traps located in the same plots. Grass cover was higher at crests and seasonal rivers than at perennial rivers and on basalts than on granites. The relationship between grass species richness and herbivore abundance or species richness was positive at crests, while that between grass species richness and herbivore species richness was negative at seasonal rivers. We found no support for controlling the dominance of grasses by herbivores in crests, but herbivore‐induced microsite heterogeneity may account for high grass species richness there. In contrast, the decrease in grass species richness with herbivore species richness at seasonal rivers indicates that the strong grazing pressure over‐rides the resistance of some species to grazing and trampling. We suggest that the relationships between grasses and herbivores may work in both directions, but the relationship is habitat‐dependent, so that in less productive environments, the effect of herbivores on vegetation prevails, while in more productive environments along rivers the effect of vegetation and water supply on herbivores is more important.

## Graphical abstract

We show the relationship between grass and herbivore species richness. We suggest that the relationships between grasses and herbivores may work in both directions, but the relationship is habitat‐dependent, so that in less productive environments the effect of herbivores on vegetation prevails, while in more productive environments along rivers, the effect of vegetation and water supply on herbivores is more important.

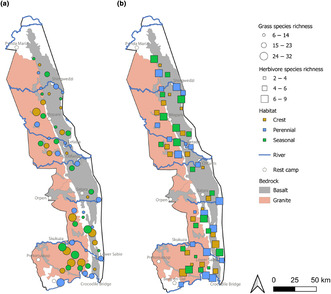

## 1 INTRODUCTION

Although the role of grazing and environmental conditions in shaping grass abundance and species richness has been investigated in several studies showing that the effect of grazing varies with the productivity of the environment (Belsky, 1992; Borer et al., 2014; Proulx & Mazumder, 1998; Ritchie & Olff, 1999, but see Koerner et al., 2018), their interaction in the savanna ecosystem at the landscape scale has not yet been studied.

The herbaceous savanna vegetation is typically characterized by the dominance of grasses that create a more or less continuous ground cover (Walker, 1987) and are an important food source for grazing herbivores. Grass abundance is primarily controlled by water and nutrient supply (Scholes, 1990; Skarpe, 1991) and by grazers and mixed‐feeders, i.e., herbivores that both browse and graze (Archibald & Hempson, 2016; du Toit, 2003; Hofmann & Stewart, 1972; Owen‐Smith, 1997). Herbivore abundance is primarily driven by local forage and water supply (Smit & Grant, 2009; Staver et al., 2019); herbivores tend to gather in areas with water sources and nutritious forage (McNaughton, 1984; Olivier & Laurie, 1974; Thrash, 1998a).

Large herbivores affect not only plant abundance but also species diversity in savannas, including that of grasses, by reducing competitively dominant species (Anderson et al., 2007; Díaz et al., 2007; Jacobs & Naiman, 2008; Olff & Ritchie, 1998; Staver & Bond, 2014). Grass diversity is expected to be highest under intermediate herbivore pressure because under high pressure, only species adapted to disturbance survive (Fenetahun et al., 2021; Smit & Grant, 2009; Thrash et al., 1993; Todd, 2006), while at low densities or when large herbivores are excluded, a few dominant grass species tend to prevail (Anderson et al., 2007; Olff & Ritchie, 1998). Grass species diversity can also be related to herbivore species diversity, which may have a two‐way explanation: (i) species‐rich grasslands attract more herbivores and (ii) more herbivores create a more heterogenous environment for a rich grass community (Jacobs & Naiman, 2008; Olff & Ritchie, 1998).

Finally, the herbivore effect on grass abundance and species richness may interact with environmental settings such as nutrients and water availability (Belsky, 1992; Milchunas et al., 1988; Veldhuis et al., 2014). Herbivory usually increases species richness in productive ecosystems while decreases were observed in low‐productive sites limited by nutrients and water (Bakker et al., 2006; Burkepile et al., 2017; Olff & Ritchie, 1998). Grazing pressure can lead to severe overgrazing and decreased grass diversity under arid and nutrient‐poor conditions (Bakker et al., 2006; Milchunas et al., 1988; Olff & Ritchie, 1998). In contrast, under high water and nutrient supply, a similar level of grazing can cause a suppression of dominant species and increase grass species richness (Belsky, 1992; Proulx & Mazumder, 1998; Ritchie & Olff, 1999). However, some comprehensive large‐scale studies only found consistent effects of herbivory, regardless of nutrient conditions. Borer et al. (2014) and Koerner et al. (2018) found that herbivore‐induced change in plant dominance was the best predictor of plant diversity worldwide, regardless of site productivity.

To explore the relationship between grasses and free‐ranging large herbivores in an African savanna, we analyzed the combined effects of grazing, habitats varying in water availability and bedrock types differing in nutrient supply on plant communities dominated by grasses in the Kruger National Park, South Africa. Given the literature cited above, we expect that (i) grass abundance will be greater on nutrient‐rich basalts than nutrient‐poor granites and will be lower in sites with more herbivores, (ii) grass species richness will be higher on granites than on basalts due to lower grass competition and lower at seasonal and perennial rivers compared to crests because of higher herbivore pressure, (iii) grass species richness will be highest under intermediate herbivore abundance and high herbivore richness, and (iv) the relationship between herbivore species richness and grass species richness will differ with regards to habitat and bedrock.

## 2 MATERIALS AND METHODS

### 2.1 Study area

The study was performed in the Kruger National Park (KNP), located in the Limpopo and Mpumalanga Provinces in northeastern South Africa. It extends 360 km from north to south and 65 km from east to west and covers almost 20,000 km^2^, which makes it one of the largest protected areas in Africa. The park has seven perennial rivers: Sabie, Olifants, Crocodile, Letaba, Shingwedzi, Luvuvhu, and Limpopo, which flow west to east (du Toit et al., 2003). The park has variable environmental conditions; altitude ranges between 140 and 780 m a.s.l, and annual precipitation between 450 and 750 mm. Bedrock is mainly acidic and nutrient‐poor in the western half of the park (granitoids) and alkalic and more nutrient‐rich in the east (mostly basalts). Grasses are represented by ~230 species in the KNP (Gertenbach, 1983), differing in their tolerance, persistence, or avoidance of grazing and in resulting growth forms (Archibald et al., 2019; Hempson et al., 2015). Grasses range from species that avoid grazing, represented by sparse short pioneer species of low nutritional value, such as *Aristida* spp. or *Pogonarthria squarrosa*, to grazing resistant species such as *Cynodon dactylon* that require grazing to avoid self‐shading, to species capable of switching to lateral growth as *Urochloa mosambicensis* and tall tussock species of a high nutritious value that can compensate for light defoliation, such as *Panicum maximum*, *Digitaria eriantha* or *Themeda triandra* (Archibald et al., 2019). There are 19 vegetation types in KNP based on a phytosociological classification (Mucina & Rutherford, 2006), of which 13 were covered by our plots; most represented were SVl3 Granite Lowveld (13 plots), SVmp4 Mopane Basalt Shrubland (12 plots), SVl5 Tshokwane‐Hlane Basalt Lowveld (10 plots), and SVmp5 Tsende Mopaneveld (9 plots; Hejda et al., 2022). The average cover of woody and grass species in our plots was 45% and 31%, respectively (see Hejda et al., 2022 for further details on vegetation cover and plant community composition).

### 2.2 Experimental plot design

**FIGURE 1:**
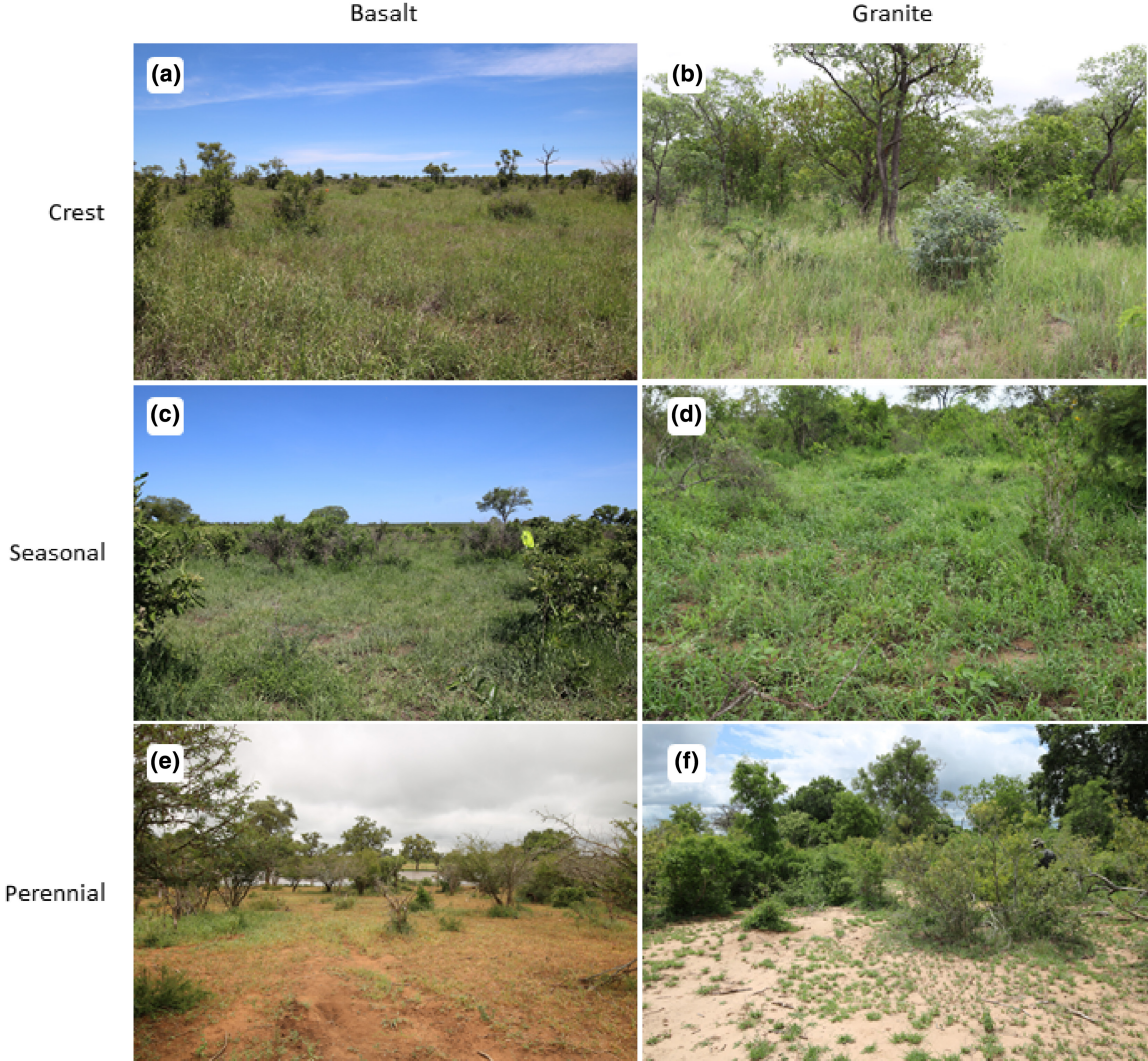
Examples of six types of open savanna based on bedrock (basalt and granite) and habitat combinations (crests, seasonal rivers, perennial rivers). (a) plot with a high grass cover at basaltic crest dominated by *Bothriochloa radicans* with *Cenchrus ciliaris*; (b) grass species‐rich plot at granitic crest with *Urochloa mosambicensis*, *Eragrostis superba*, *Melinis repens* and *Trichoneura grandiglumis*; (c) plot at a seasonal river on basalt with abundant *Digitaria eriantha*, *Panicum coloratum* and *Panicum maximum*; (d) plot at a seasonal river on granite with *Urochloa mosambicensis*, *Panicum maximum* and *Eragrostis cilianensis*; (e) plot at a perennial river on basalt with a sparse vegetation with *Eragrostis rigidior*, *Urochloa mosambicensis* and *Tragus berteronianus*; (f) grass species‐poor plot at a perennial river on basalts with *Sporobolus nitens* and *Tragus berteronianus*.

**FIGURE 2:**
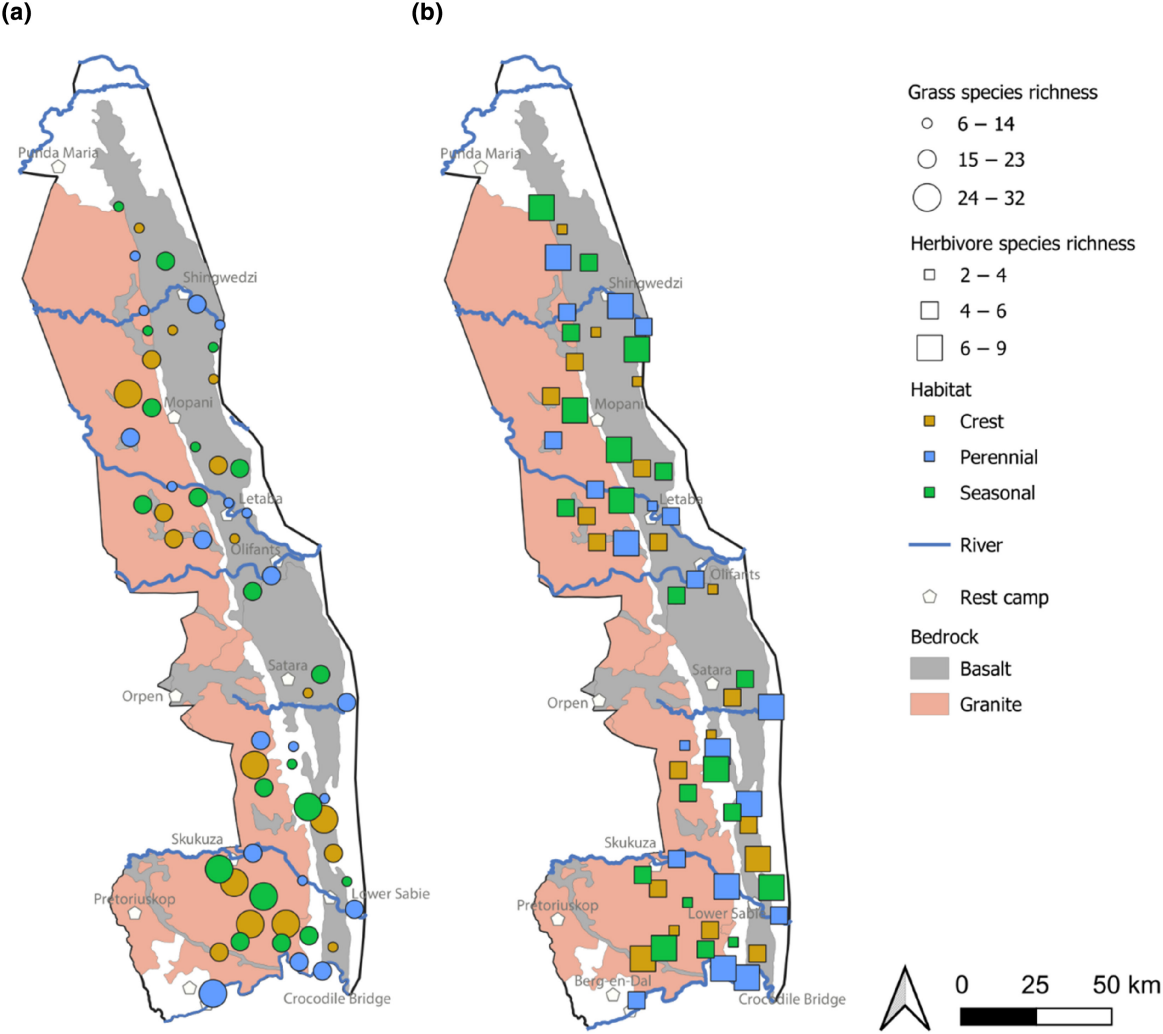
Map showing grass (a) and herbivore species richness (b) in three different habitats on two bedrocks. Symbol size represents species richness within each group.

Data were collected within the MOSAIK (Monitoring Savanna Biodiversity in the Kruger National Park) project, which explores the patterns and interactions among plant, insect, bird, and mammal communities (see Hejda et al., 2022). Data were collected along a gradient of water availability and on two types of bedrock. Water supply was accounted for by locating the plots in three habitats: (i) near a perennial river or another permanent water source, such as artificial water points or dams, (ii) near a seasonal river with a lack of water during dry periods, and (iii) on dry crests, at least 5 km from any source of water (Figure 1). These habitat types affect surface water availability for animals as well as soil moisture for deep‐rooting perennial species. The plots were grouped into triplets by habitats, with plots within the triplet being closer to each other (~7–13 km apart) than to other plots outside the triplet. The plots were located on two contrasting bedrocks, basalt and granite, which weather into soils that differ in nutrients, water retention capacity, and texture (Mucina & Rutherford, 2006; Venter et al., 2003). We established 60 permanent plots of 50 × 50 m that were equally distributed among the three habitats (20 in each) and two bedrocks (30 in each, see Figure 2). As we focused on the shrubby savanna, we placed our plots outside the riparian gallery forests, and the presence of trees in the plots was generally very low, if any.

### 2.3 Plant data

Vegetation was sampled in the plots during the peak growing season (see also Hejda et al., 2022; Pyšek et al., 2020). Surveys were done during two rainy seasons, from 16 January to 4 February 2019 (33 plots) and 17 January to 3 February, 2020 (27 plots); the plots sampled in each year were equally stratified among habitats and bedrocks. All plant species (for simplicity, intraspecific taxa such as subspecies are further also referred to as ‘species’) were recorded in each 2500 m^2^ plot, separately for the shrub and herb layer, and their abundances were visually estimated using the Braun‐Blanquet cover‐abundance seven‐grade scale (Mueller‐Dombois & Ellenberg, 1974). To quantify the occurrence of species in the plots, the Braun‐Blanquet scores were transformed to percentage values as follows: 5 = 87.5%, 4 = 62.5%, 3 = 37.5%, 2 = 15%, 1 = 2.5%, + = 1.0%, *r* = .02% (van der Maarel, 1979).

For each plot, we calculated (i) grass species richness, i.e., the total number of grass species recorded in the plot and (ii) grass cover as the sum of the ground covers of all grass species present in the plot; the grass cover ranged between 1% and 103% (because leaf layers may overlap across species, summary values over 100% are possible). The nomenclature of plant species follows van Oudtshoorn (2018).

### 2.4 Herbivore data

The presence of animal species was recorded by camera traps located in the same plots where the vegetation sampling was performed (one in each plot, *n* = 60). Bushnell Essential E3 camera traps with low glow IR flash were used to collect data on large herbivores. The camera traps were set to take three consecutive images once the camera was triggered to make species identification easier, but later, duplicates were removed for the analysis. The following image was considered when the camera was triggered again. When more individuals of the same species occurred in one image, we considered them a single record; this approach was used to standardize the data and avoid bias potentially caused by fluctuations in the lengthy occurrence of large herds. We assumed that the impact of herbivores on grasses manifests by a combination of three factors: (i) the abundance of herbivores, (ii) the duration of their presence, and (iii) their activity – only animals that were moving were recorded. All three factors were captured by the camera settings and contributed to the increase in the number of records. In this article, we use data from a total of 140 days from both dry (June–August 2018) and rainy (December–February 2019) seasons.

From this camera‐based dataset, we extracted records of large herbivores (elephants, equids, rhinoceroses, hippos and bovids) that are reported to influence grass species composition (Cumming, 1982; du Toit, 2003). We considered both grazers and mixed feeders, i.e., species that feed on grass (Table 1); for simplicity, we use the term ‘herbivore’ for both groups. The feeding strategy (grazer or mixed‐feeder) was taken from Estes (2012). For each plot, we calculated (i) herbivore species richness, expressed as the total number of herbivore species recorded over the monitoring period, and herbivore species abundances, corresponding to the total number of records of a given species. The sum of records of all grazing species (Table 1) served as a proxy for (ii) herbivore abundance. We are aware that the term ‘abundance’ as we use it does not correspond to the exact numerical abundance of species populations per plot, but we suggest that it is an informative measure of the total herbivore pressure in a plot. To account for differences in herbivore size, we also estimated the total herbivore weight per plot, which was calculated as species abundance × mean species weight summed over all species. Species weights were taken from Kingdon et al. (2013) and Kingdon and Hoffmann (2013a, 2013b) and calculated as a mean of the ranges given for male and female weight (Appendix S1). However, as most records belonged to a few species, this measure was strongly correlated with herbivore abundance (*r* = .65, see Appendix S2 for mutual correlations between predictor and response variables) and we therefore decided not to use it. Similarly, we did not distinguish between grazers and browsers as herbivore abundance and grazer abundance were strongly correlated (*r* = .59); the same was true for herbivore abundance and grazer weight (*r* = .68).

**TABLE 1:** Overview of herbivore (grazers and mixed‐feeders) species recorded by camera traps in 60 plots in the Kruger National Park (see text for details).

Common name	Scientific name	Feeding strategy	Number of plots	Number of records
Impala	*Aepyceros melampus*	Mixed	55	38,154
Elephant	*Loxodonta africana*	Mixed	59	11,644
Buffalo	*Syncerus caffer* subsp. *caffer*	Grazer	34	4616
Waterbuck	*Kobus ellipsiprymnus*	Grazer	26	3124
Common duiker	*Sylvicapra grimmia*	Mixed	40	2750
Zebra	*Equus quagga*	Grazer	44	2133
Hippo	*Hippopotamus amphibius*	Grazer	22	1785
Wildebeest	*Connochaetes taurinus*	Grazer	18	1379
Nyala	*Tragelaphus angasii*	Mixed	12	1034
White rhino	*Ceratotherium simum* subsp. *simum*	Grazer	16	464
Tsessebe	*Damaliscus lunatus* subsp. *lunatus*	Grazer	3	8
Sable antelope	*Hippotragus niger*	Grazer	1	7
Eland	*Taurotragus oryx*	Mixed	1	2

*Note*: Feeding strategy was taken from Estes (2012). Number of plots = number of plots in which the species occurred at least once; number of records = total number of records from all plots. Species are arranged by the number of records from camera traps.

### 2.5 Data analysis

#### 2.5.1 Univariate analyses: grass cover and species richness

The effects of herbivore abundance and species richness, habitat, bedrock, and all their mutual two‐way interactions (excluding the interaction between herbivore abundance and herbivore species richness) on grass cover and species richness (response variables) were tested using linear and generalized linear mixed‐effect models – LMM and GLMM (Table 1, see Appendix S3 for model formulas). Data on abundance and species richness of herbivores were merged for both seasons, as separate models for dry, wet and both seasons merged gave similar results (not shown). Triplet identity, which reflected the spatial clustering of the plots, was set as a random variable. For grass cover and species richness, we used LMMs with normal distribution; the response variable grass species richness was square‐root transformed to improve normality and homogeneity of variance (see Appendix S4 for the verification of normality and heteroskedasticity in grass species richness). Herbivore abundance and richness were standardized by the scale function in R, i.e., the mean was subtracted from each value and then divided by the standard deviation to unify the range of both predictors. To test possible non‐linear effects of herbivore abundance and herbivore species richness, we added their quadratic terms to the models; however, as they were not significant, they were not included in the final models. The significance of the terms in LMM and GLMM models was obtained via the *Anova* function from the “car” package (Fox & Weisberg, 2019). We used a type II ANOVA where the main effects are interpreted, controlling for their overlap with other main effects and interactions. Regression lines and estimates were created using simplified LM and GLM models that contained only the predictor and response variables shown on the given graph (Figures 6 and 7). The differences among individual habitats in the simplified models with just a single predictor and a single response variable were further tested by the post‐hoc pairwise comparison of estimated marginal means with Tukey adjustment for multiplicity (Lenth, 2021). In addition to the R‐base packages, we further used the package “nlme” (Pinheiro et al., 2017) and “lme4” (Bates et al., 2015) for fitting the linear mixed‐effect models and generalized mixed‐effect models, respectively. Package “emmeans” was used for subsequent multiple comparisons among significant terms (Lenth, 2021). Graphs were plotted using the “tidyverse” (Wickham et al., 2019), and “ggpubr” (Kassambara, 2020) packages. All computations were done in the program R 4.2.0 (R Core Team, 2022).

#### 2.5.2 Multivariate analyses: grass community composition

The aim was to analyze the effect of herbivores on the grass community composition. Percentages of grass species covers (proxies for abundance) were response variables, and (i) the herbivore abundance and the herbivore species richness or (ii) the abundance of individual herbivore species were predictors (Table 1). To account for the effect of habitat, bedrock, and spatial arrangement given by the mutual position of permanent plots, we set them as covariables in all analyses. Spatial effects (autocorrelation of plots) were identified by the PCNM analysis (ter Braak & Šmilauer, 2012), where GPS coordinates represented the plot position, from which a matrix of spatial PCoA vectors was calculated; here, we used the scores of the first three PCoA vectors as spatial covariables. Because plots were grouped in triplets in the field, we used a hierarchical split‐plot design, where triplets were set as whole plots, while plots within a triplet represented split plots. Both triplets and plots within triplets were permuted freely, and the significance was tested by Monte‐Carlo permutation tests. We further tested the effect of herbivore abundance on the grass community composition at crests to explore the possible dominance‐control mechanisms; no covariables or hierarchical arrangements were used in this analysis. In addition to the effect of herbivores, we also tested the differences in grass species composition between bedrocks (basalt and granite); the triplets were set as covariables and permutation scheme was set up similarly as in the main analyses testing relationship between plant composition and herbivores. All multivariate analyses were performed in Canoco 5 software (Šmilauer & Lepš, 2014). In both univariate and multivariate analyses, we interpreted p‐values between .05 and .1 as marginally significant.

## 3 RESULTS

### 3.1 Herbivore load by habitat

**FIGURE 3:**
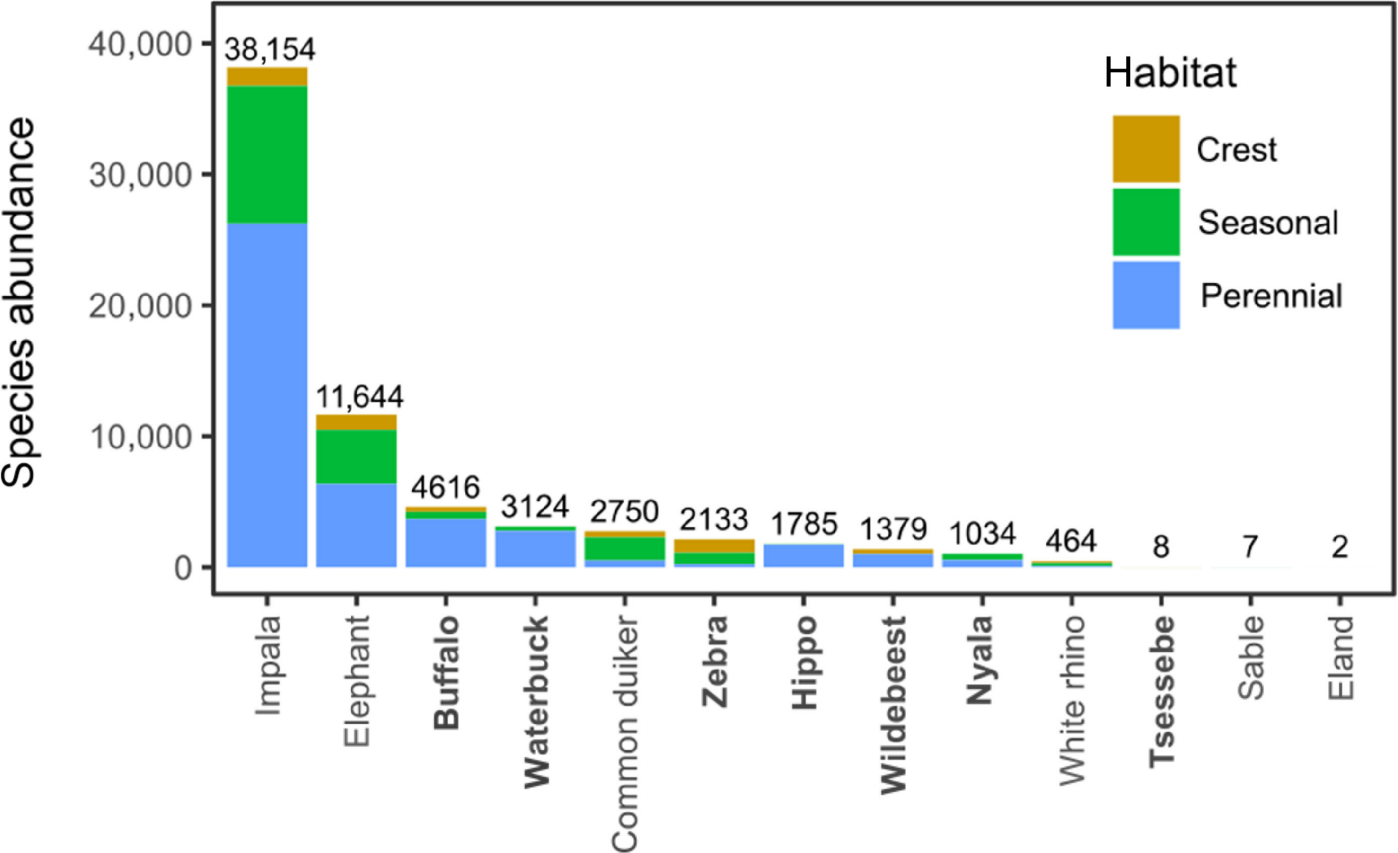
The total number of records across all plots of herbivores (grazers in bold, mixed‐feeders in plain font), with habitats (perennial rivers, seasonal rivers, crests) indicated by different colors. Altogether, we recorded 67,100 animals in 60 plots over 140 days (June 2018 to February 2019). Tsessebe, sable and eland were not included in further analyses because of the low number of records.

Of the 67,100 records of herbivores (grazers and mixed‐feeders) captured by camera traps over the study period, the majority were from perennial rivers (43,195 records; 64.4%; of which 18,994 were recorded in the rainy season), while seasonal rivers experienced an intermediate herbivore load (18,961 records; 28.2%; 7254 in the rainy season), and the fewest herbivores were recorded at crests (4944 records; 7.4%; 2996 in the rainy season). We recorded eight species of grazers (buffalo, hippo, tsessebe, waterbuck, white rhino, wildebeest, sable antelope, and zebra) and five mixed‐feeders (common duiker, eland, elephant, impala, nyala; Table 1). The most common herbivore was impala, accounting for more than half of all records (38,154 records; 54.1%), followed by elephant (11,644 records; 16.5%) and buffalo (4616; 6.6%). The abundances of all herbivore species across habitats are shown in Figure 3. Species that occurred in the greatest number of plots were elephant (58 plots, i.e., 98.3% of the total of 60 monitored), impala (56 plots; 93.3%), and buffalo (52 plots; 86.7%). Species with only a few records (tsessebe = 8, sable antelope = 7, and eland = 2) were excluded from analyses (Table 1).

### 3.2 Grass cover

**FIGURE 4:**
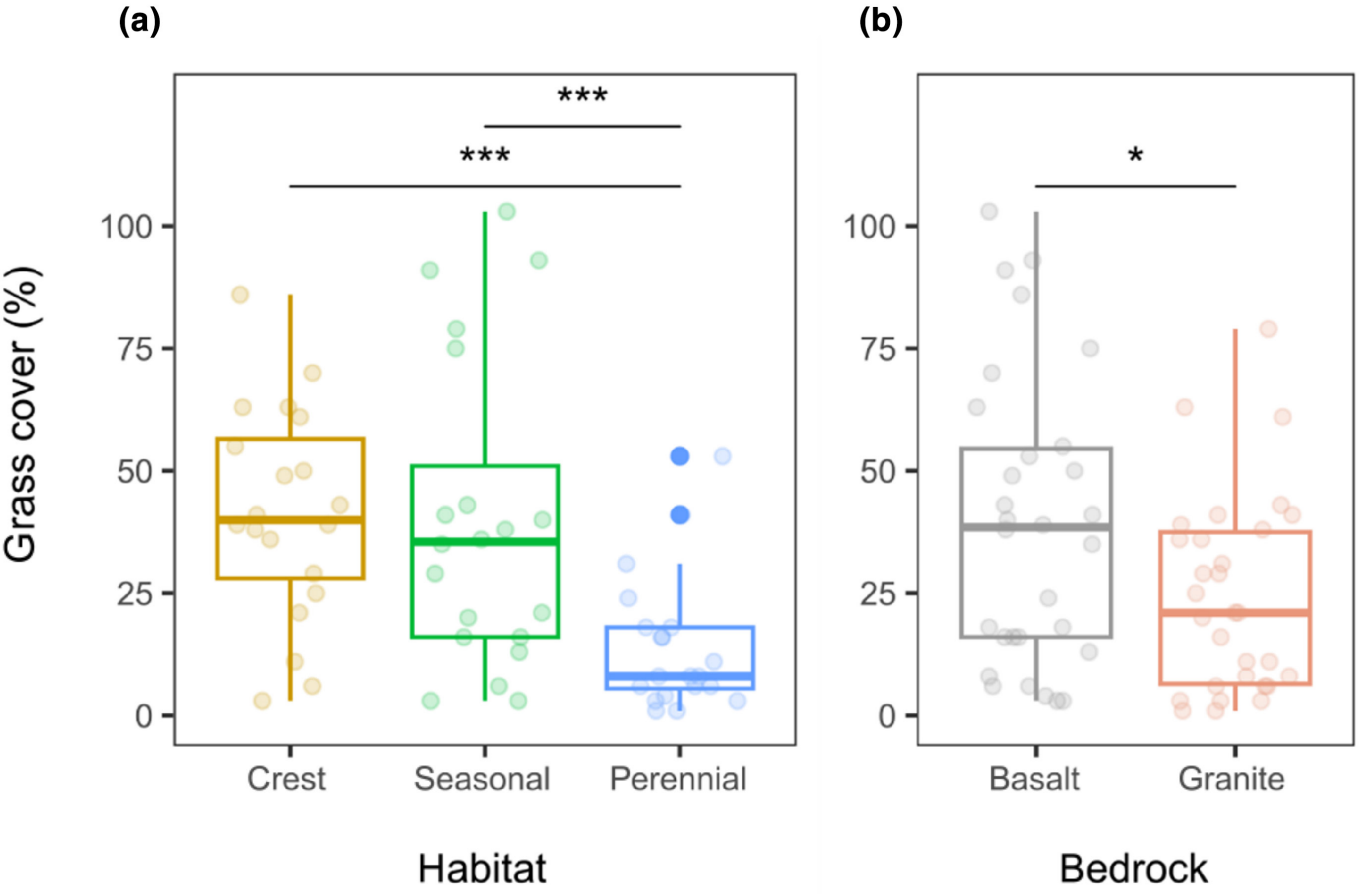
Differences in total grass cover per plot, expressed as the sum of covers of all grass species recorded, between habitats (a) and bedrocks (b). Transparent points represent grass cover for each habitat (*n* = 20) or bedrock (*n* = 30), solid points (only for perennial rivers) represent outliers. All points show raw data. Significance *p*: . (.1–.05), * (.05–.01), *** (<.001), non‐significant values are not shown.

We recorded 99 grass species that, on average, reached the total cover, summed across all grass species in a plot of 31.9%. There were significant differences in grass cover among habitats and bedrock types (Table 2). Grasses had a significantly higher cover at crests (*p* < .001, mean value of 41.5%) and at seasonal rivers (*p* < .001, 40.1%) than at perennial rivers (14.1%; Figure 4a) and on basalts (*p* = .041, 39.2%) than on granites (24.5%; Figure 4b). We found no significant relationship between herbivore species richness and grass cover, either as a main effect or in interaction with bedrock or habitat (Table 2).

**TABLE 2:** Results of LMM and GLMM models for grass cover and grass species richness, respectively.

Predictors	Grass cover			Grass species richness		
	Chi	*df*	*p*	Chi	*df*	*p*
Herbivore abundance (HA)	1.12	1	.290	0.01	1	.942
Herbivore species richness (HSR)	0.10	1	.747	0.01	1	.952
Habitat (H)	17.93	2	**<.001**	4.56	2	.102
Bedrock (B)	3.67	1	.055	3.93	1	**.047**
HA × H	0.04	2	.982	5.14	2	.076
HA × B	0.24	1	.622	0.14	1	.709
HSR × H	0.62	2	.733	6.06	2	**.048**
HSR × B	0.22	1	.639	1.06	1	.303
H × B	2.88	2	.237	2.95	2	.229

*Note*: List of predictors: herbivore abundance; herbivore species richness; habitat = location of plots at perennial, seasonal rivers or crests; bedrock = location of plots on granite or basalt. Significant results (*p* < .05) are marked in bold, marginally significant (*p* = .1–.05) are underlined. Herbivore abundance and richness were scaled in both models.

### 3.3 Grass species richness

**FIGURE 5:**
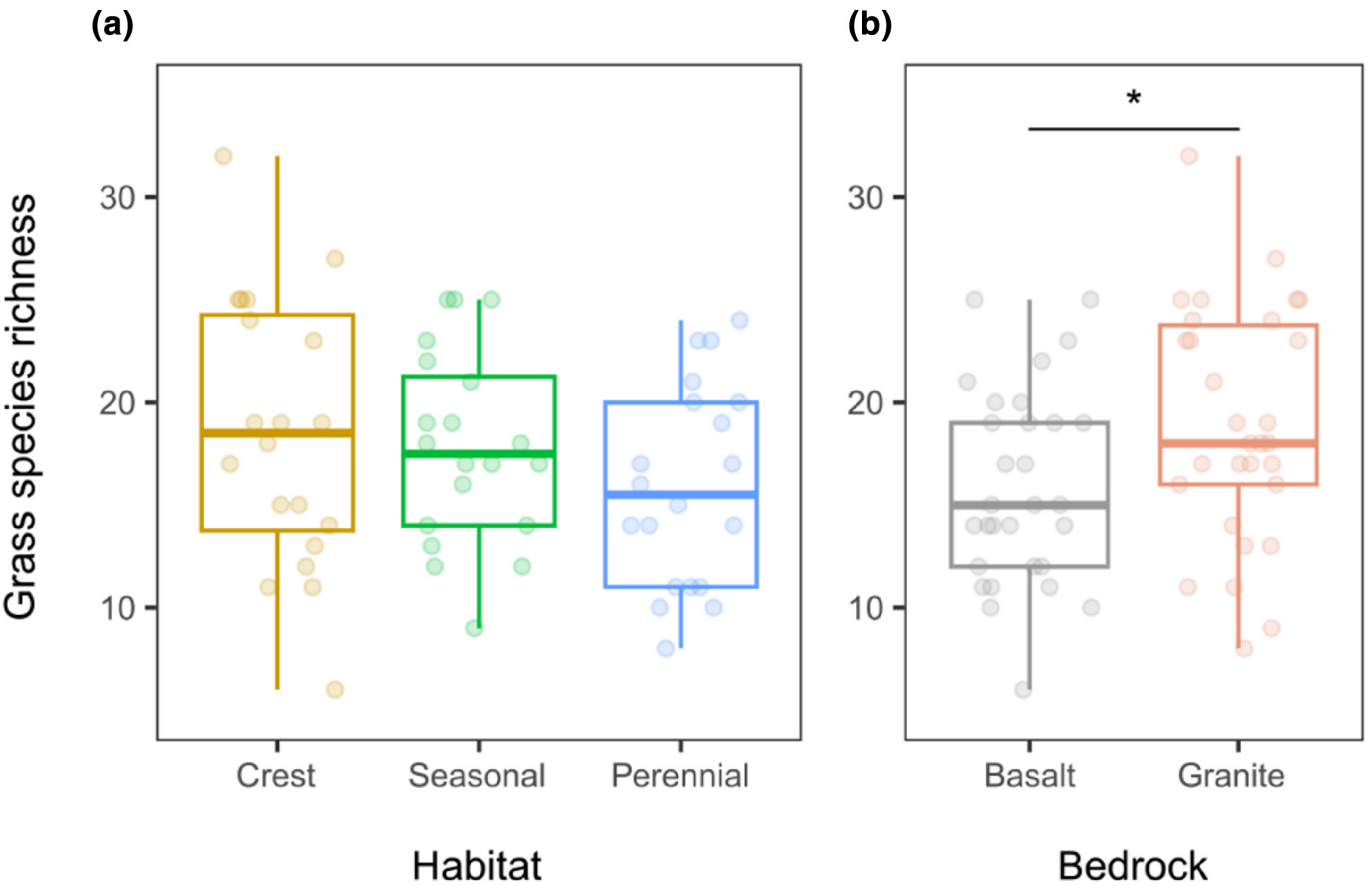
Differences in grass species richness per plot, expressed as the sum of all grass species recorded, between habitats (a) and bedrocks (b). Transparent points represent grass cover for each habitat (*n* = 20) or bedrock (*n* = 30), solid points (only for perennial rivers) represent outliers. All points show raw data. Significance *p*: * (.05–.01), non‐significant values are not shown.

Grass species richness did not differ significantly among habitats (*p* = .102, Figure 5a and Table 2), but differed between bedrocks; there were more species on granites than on basalts (*p* = .047, Figure 5b). The highest grass species richness was recorded on granitic crests; however, the interaction of bedrock and habitat was not significant.

**FIGURE 6:**
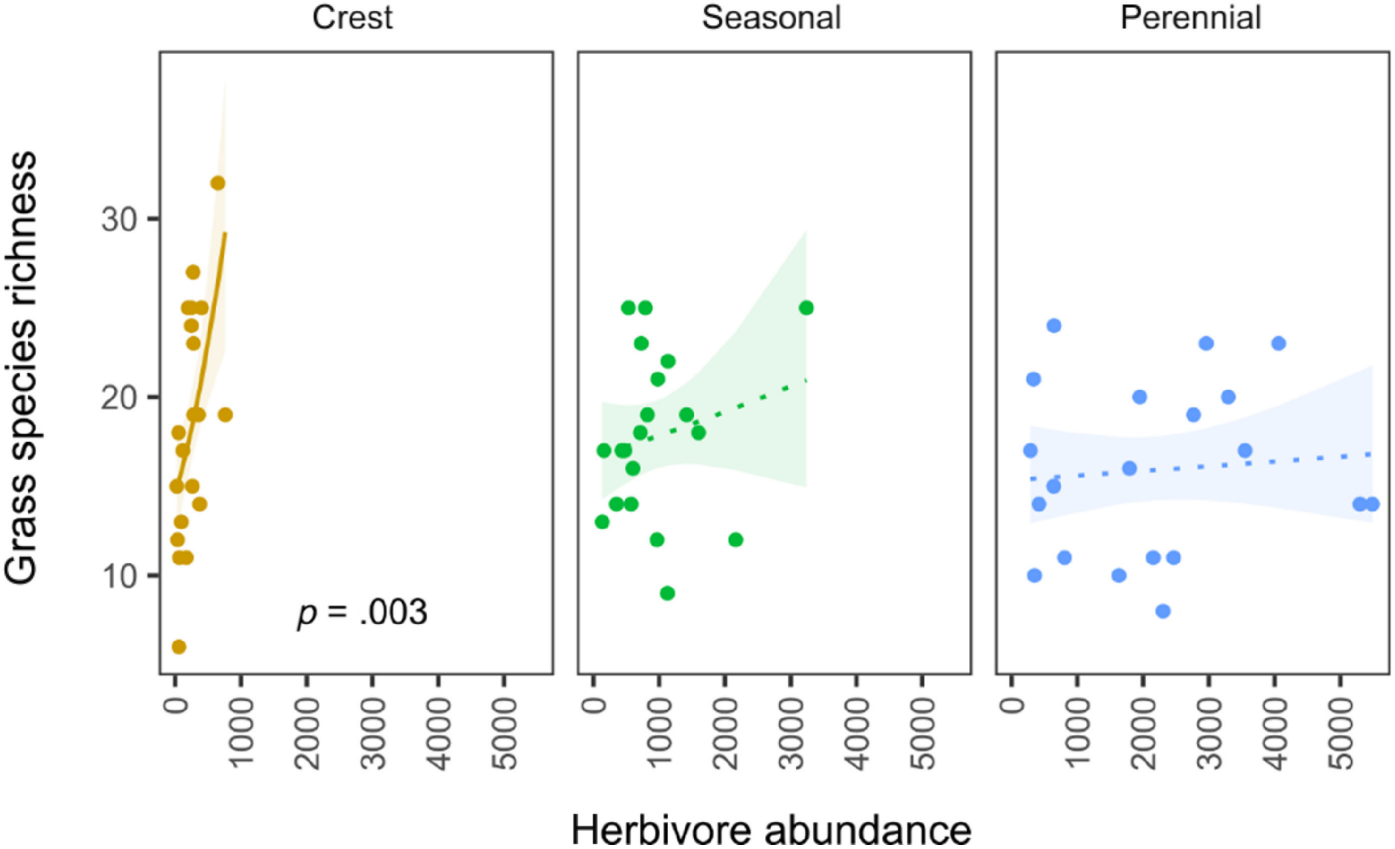
Relationship between grass species richness and herbivore abundance shown for different habitats. The regression lines show fit of linear models. Shaded areas show 95% confidence intervals, nonsignificant relationships for seasonal and perennial rivers are indicated by dotted lines. All points show raw data.

Neither herbivore abundance nor species richness significantly explained grass species richness as the main effect (Table 2). However, interactions with habitat were significant for herbivore species richness (*p* = .048) and marginally significant for herbivore abundance (*p* = .076; Table 2). Grass species richness increased steeply with herbivore abundance on crests (*p* = .079, Figure 6); the slope of this relationship seemed to decrease with water availability, but the herbivore abundance vs. grass species richness relationship was significant at neither seasonal nor perennial rivers (*p* = .345 and .692, respectively, see Figure 6).

**FIGURE 7:**
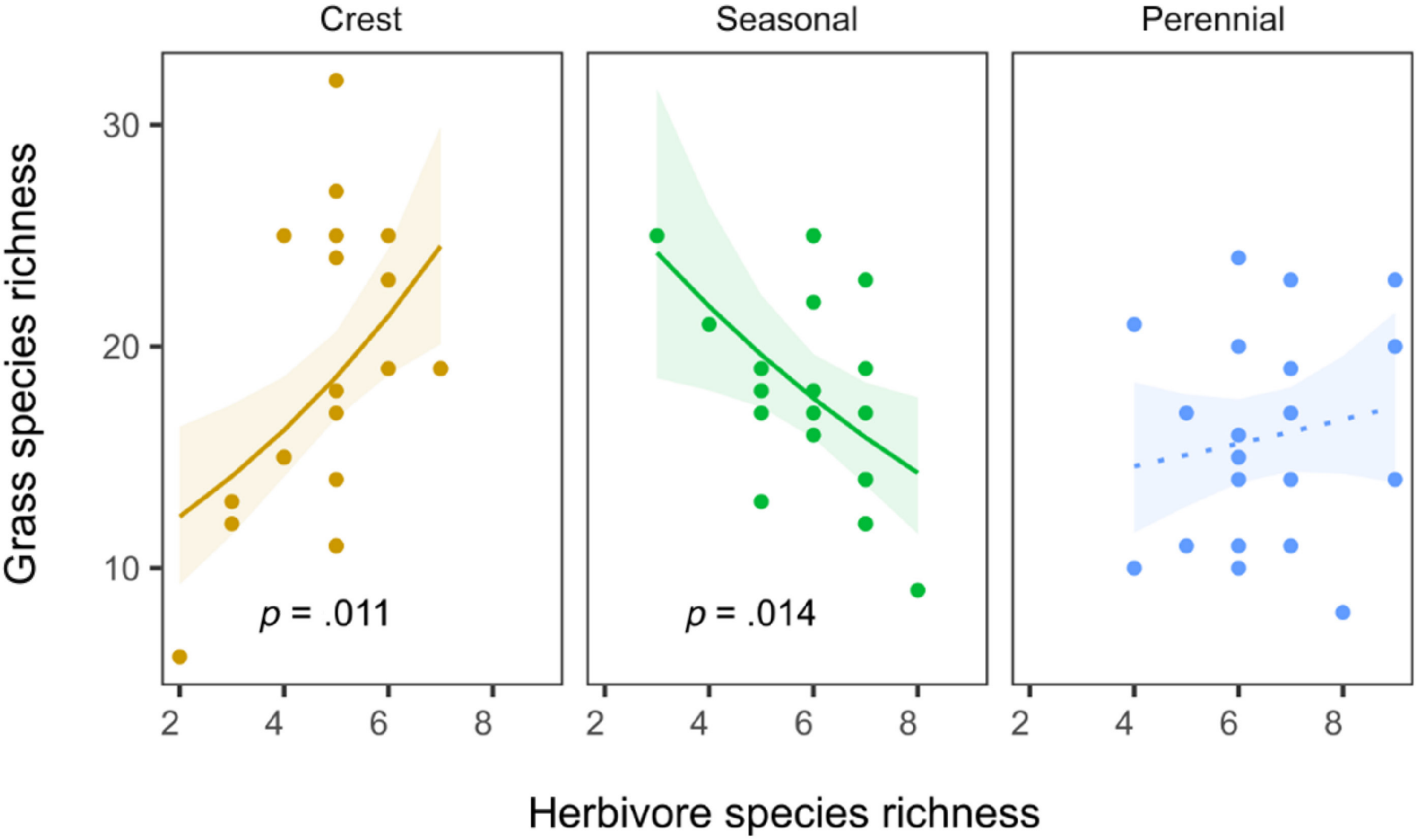
Relationship between grass species richness and herbivore species richness shown for different habitats. The regression lines show the fit of generalized linear models. Shaded areas show 95% confidence intervals, nonsignificant relationships for perennial rivers are indicated by dotted lines. All points show raw data.

Grass species richness was significantly positively related to herbivore species richness at crests (*p* = .011) and negatively at seasonal rivers (*p* = .014); the relationship was nonsignificant at perennial rivers (*p* = .469, Figure 7).

### 3.4 Grass community composition

The most common grass species were *Panicum maximum*, occurring in 54 plots (90% of the total number sampled), *Brachiaria deflexa* (52 plots; 86.7%), and *Tragus berteronianus* (49 plots, 81.7%; see Table 3). The most abundant in terms of the mean cover per plot were *Bothriochloa radicans* (14.1%, present in 20 plots), *Hyparrhenia hirta* (6.6% in 8 plots), and *Themeda triandra* (5.8% in 21 plots).

**TABLE 3:** Ten most common grass species according to their frequency, expressed as their occurrence in plots.

Species	Life history	Grazing value	Number of plots	Mean cover (%)
Ranked by frequency				
*Panicum maximum*	Perennial	High	54	5.0 ± 11.5
*Brachiaria deflexa*	Annual	Medium	52	2.5 ± 6.1
*Tragus berteronianus*	Annual	Low	49	0.9 ± 2.3
*Urochloa mosambicensis*	Weak perennial	Medium	43	4.5 ± 10.6
*Aristida adscensionis*	Annual	Low	41	0.7 ± 2.4
*Eragrostis superba*	Weak perennial	Medium	36	2.6 ± 7.3
*Digitaria eriantha*	Perennial	High	35	1.3 ± 3.5
*Enneapogon cenchroides*	Weak perennial	Medium	32	3.1 ± 9.4
*Cenchrus ciliaris*	Perennial	High	31	3.4 ± 8.1
*Heteropogon contortus*	Perennial	Medium	30	0.3 ± 0.8
Ranked by cover				
*Bothriochloa radicans*	Perennial	Low	20	14.1 ± 20.2
*Hyparrhenia hirta*	Perennial	Medium	8	6.6 ± 13.5
*Themeda triandra*	Perennial	High	21	5.8 ± 13.3
*Panicum maximum*	Perennial	High	54	5.0 ± 11.5
*Panicum deustum*	Perennial	Medium	10	4.5 ± 11.6
*Urochloa mosambicensis*	Weak perennial	Medium	43	4.5 ± 10.6
*Bothriochloa insculpta*	Annual	Medium	10	4 ± 11.8
*Cenchrus ciliaris*	Perennial	High	31	3.4 ± 8.1
*Setaria incrassata*	Perennial	High	12	3.4 ± 10.8
*Enneapogon cenchroides*	Weak perennial	Medium	32	3.1 ± 9.4

*Note*: The number of plots (*n* = 60) in which the species was recorded and its mean cover within them (mean ± SD) are given. Life history data and grazing values were taken from van Oudtshoorn (2018), see text for details. Species were classified as annuals if they grow for one season, as weak perennials if they grow for two to five seasons, and as perennials if they persist for longer than five seasons.

Using multivariate analyses with habitat, bedrock, and spatial arrangements as covariables, we did not find any significant effects of herbivore abundance and species richness on grass species' covers (*F* = 0.9, *p* = .66). Similarly, there was no significant effect of the abundance of particular herbivore species on grass species covers (*p* = .162); only when testing individual species by the forward selection, the effect of hippo (*F* = 2.9, *p* = .012) and scrub hare (*F* = 1.6, *p* = .092) had significant and marginally significant effects, respectively. We also found no difference in grass community composition between bedrocks (*F* = 1.3, *p* = .144) indicating, e.g., a greater dominance of some species on basalt or granite. Lastly, we found no effect of herbivore abundance or species richness on grass community composition (*F* = 1.1, *p* = .356 and *F* = 1.1, *p* = .375, respectively), showing possible control of dominant grasses by herbivores.

## 4 DISCUSSION

### 4.1 Grass cover

In our study system, grass cover decreased with increasing water availability towards perennial rivers, where most animals were recorded. This corresponds with the results of Olivier and Laurie (1974), who found that grass cover increased from 34% to 71% over one kilometer from the river, and grazing intensity (measured along a transect as the percentage of grazed vegetation) declined from 86% at 20 m from the river to 35% at 880 m apart at the Mara River in Tanzania. A similar pattern was found at artificial watering points, where herb and shrub cover were lowest near water and increased with distance from the water source (Smit & Grant, 2009; Thrash, 1998b; Thrash et al., 1993; Todd, 2006). In terms of bedrock, grass cover was two times higher on nutrient‐rich clayey soils derived from basalts than on sandy soils on granites, which corresponds with the results of Dye and Spear (1982), who found greater grass biomass on clayey soils in Zimbabwean savanna systems.

### 4.2 Effect of bedrock on grass species richness

Grass species richness was higher on granites than on basalts. However, we did not confirm our hypothesis that this is because of the dominance of competitively strong grasses on nutrient‐rich basalts. Our results showed no difference in the grass community composition, considering species' covers, between basalt and granite, which was consistent with our previous study for all herbs (Hejda et al., 2022). Therefore, it seems that low grass species richness on basalts is rather due to the extremity of the environment (Zambatis, 2003). The specific feature of the clayey soils on basalts, with the quick water runoff, is rapid desiccation, in contrast to more stable sandy soils that carry over the soil moisture stored in the subsoil from one year to another (Dye & Spear, 1982). Large moisture fluctuations in clayey soils may aggravate the establishment and survival of some species, leading to the persistence of fewer specialized species typical of clayey soils, such as *Panicum coloratum* or *Bothriochloa radicans*, and, in turn, lower species diversity (Dye & Spear, 1982).

### 4.3 Grass species richness: Interaction of habitat, herbivore abundance, and herbivore species richness

Although there was no overall effect of herbivore abundance (and species richness) on grass species richness, there was a significant interaction with habitat. Grass species richness steeply increased with herbivore abundance or herbivore species richness in crests. This can have bidirectional explanation: (i) herbivore abundance or species richness may be driven by grass richness as more species provide broader food offer (Burkepile et al., 2017; Kallay & Cohen, 2008; Malard et al., 2020) or/and (ii) herbivores may support grass richness by acting as a dominance control mechanism, suppressing potentially dominant species, and increasing microsite heterogeneity (Chaneton & Facelli, 1991; Jacobs & Naiman, 2008; Koerner et al., 2018; La Plante & Souza, 2018; Ritchie & Olff, 1999).

We found no support for herbivore dominance control in crests; there were no significant changes in grass species composition along a gradient of herbivore abundance or species richness. An experimental approach with manipulated herbivore exclusion would be required (Bakker et al., 2006) to obtain deeper insight into mechanisms that are in play. In addition, specific herbivore activities, such as digging or wallowing creating soil disturbations, or defecation and urination causing local nutrient enrichment that we have not studied may also affect microsite heterogeneity and plant species richness.

It is impossible to solve this dilemma of cause and consequence using comparative data, and even exclosure experiments tend to give ambiguous results (Chikorowondo et al., 2017; Fenetahun et al., 2021; Li et al., 2017). Moreover, it is likely that both mechanisms with opposite directions are at play with differing importance depending on the specific environmental settings and composition of biotic communities. In general, we suggest that the relationship between grasses and herbivores may work in both directions, but it is habitat‐dependent, so in the less productive environment, the effect of herbivores on vegetation prevails (documented, e.g., by Ritchie & Olff, 1999, Thrash, 1998b, Thrash et al., 1993), while in more productive environments along rivers, the effect of vegetation and water supply on herbivores is more important (Jacobs & Naiman, 2008).

### 4.4 Management implications

Our results suggest that it is necessary to critically assess local environmental conditions in protected areas that aim to support grass species richness. Depending on the context, the same herbivore abundance generates different outcomes, and both overgrazing as well as low grazing pressure may lead to grasslands dominated by a few species. Grass species richness is important not only per se but also because more grass species form a more heterogeneous environment that supports the diversity of animals and other plant species. Such a mosaic of species‐rich grasslands offers a broad forage supply and contains species of different successional stages, making them more resistant to different scenarios, such as fluctuation of grazing pressure, fire, or drought.

## AUTHOR CONTRIBUTIONS

**Jan Čuda:** Data curation (equal); formal analysis (lead); investigation (equal); visualization (lead); writing – original draft (lead); writing – review and editing (lead). **Klára Pyšková:** Conceptualization (equal); data curation (equal); investigation (equal); methodology (equal); writing – original draft (equal); writing – review and editing (equal). **Martin Hejda:** Conceptualization (equal); data curation (equal); formal analysis (equal); investigation (equal); methodology (equal); writing – original draft (equal); writing – review and editing (equal). **Llewellyn C. Foxcroft:** Conceptualization (equal); methodology (equal); project administration (equal); writing – original draft (equal); writing – review and editing (equal). **Sandra MacFadyen:** Conceptualization (equal); writing – review and editing (equal). **David Storch:** Conceptualization (equal); methodology (equal); writing – original draft (equal); writing – review and editing (equal). **Robert Tropek:** Conceptualization (equal); methodology (equal); writing – original draft (equal); writing – review and editing (equal). **Guin Zambatis:** Supervision (equal); writing – review and editing (equal). **Petr Pyšek:** Conceptualization (lead); funding acquisition (lead); investigation (equal); methodology (equal); project administration (lead); writing – original draft (equal); writing – review and editing (equal).

## Supporting information


Appendix S1.–S4.

Data S1.

## Data Availability

The data that support the findings of this study are available in the [Link], [Link].
