# Supplementary material for: Habitat modifies the relationship between grass and herbivore species richness in a South African savanna
Source: Ecol Evol. 2024 Apr 15;14(4):e11167. doi: 10.1002/ece3.11167 (PMC11016939; doi:10.1002/ece3.11167)
Supplement: Supplementary file 1 — Appendix S1.–S4. [file ECE3-14-e11167-s001.docx]

**Appendix S1.**

Overview of grazer (including mixed-feeder) species recorded by camera traps in 60 plots in the Kruger National Park (see text for details). Feeding strategy was taken from Estes (2012). Mean weight = mean of the male and female weight; number of plots = number of plots in which the species occurred at least once; number of records = total number of records from all plots. Species are arranged alphabetically by their common name. Data on animal body weight were taken from Kingdon et al. (2013)^1^, Kingdon & Hoffmann (2013a)^2^ and Kingdon & Hoffmann (2013b)^3^.

| Common name | Scientific name | Feeding strategy | Mean weight (kg) | Number of plots | Number of records | Ref. |
| --- | --- | --- | --- | --- | --- | --- |
| buffalo | *Syncerus caffer* subsp. *caffer* | grazer | 648 | 34 | 4616 | 3 |
| common duiker | *Sylvicapra grimmia* | mixed | 19 | 40 | 2750 | 3 |
| eland | *Taurotragus oryx* | mixed | 561 | 1 | 2 | 3 |
| elephant | *Loxodonta africana* | mixed | 4035 | 59 | 11644 | 1 |
| hippo | *Hippopotamus amphibius* | grazer | 1400 | 22 | 1785 | 3 |
| impala | *Aepyceros melampus* | mixed | 58 | 55 | 38154 | 3 |
| nyala | *Tragelaphus angasii* | mixed | 87 | 12 | 1034 | 3 |
| white rhino | *Ceratotherium subsp. simum simum* | grazer | 2000 | 16 | 464 | 2 |
| sable antelope | *Hippotragus niger* | grazer | 228 | 1 | 7 | 3 |
| tsessebe | *Damaliscus lunatus* | grazer | 129 | 3 | 8 | 3 |
| waterbuck | *Kobus ellipsiprymnus* | grazer | 209 | 26 | 3124 | 3 |
| wildebeest | *Connochaetes taurinus* | grazer | 275 | 18 | 1379 | 3 |
| zebra | *Equus quagga* | grazer | 242 | 44 | 2133 | 2 |

**Appendix S2**.
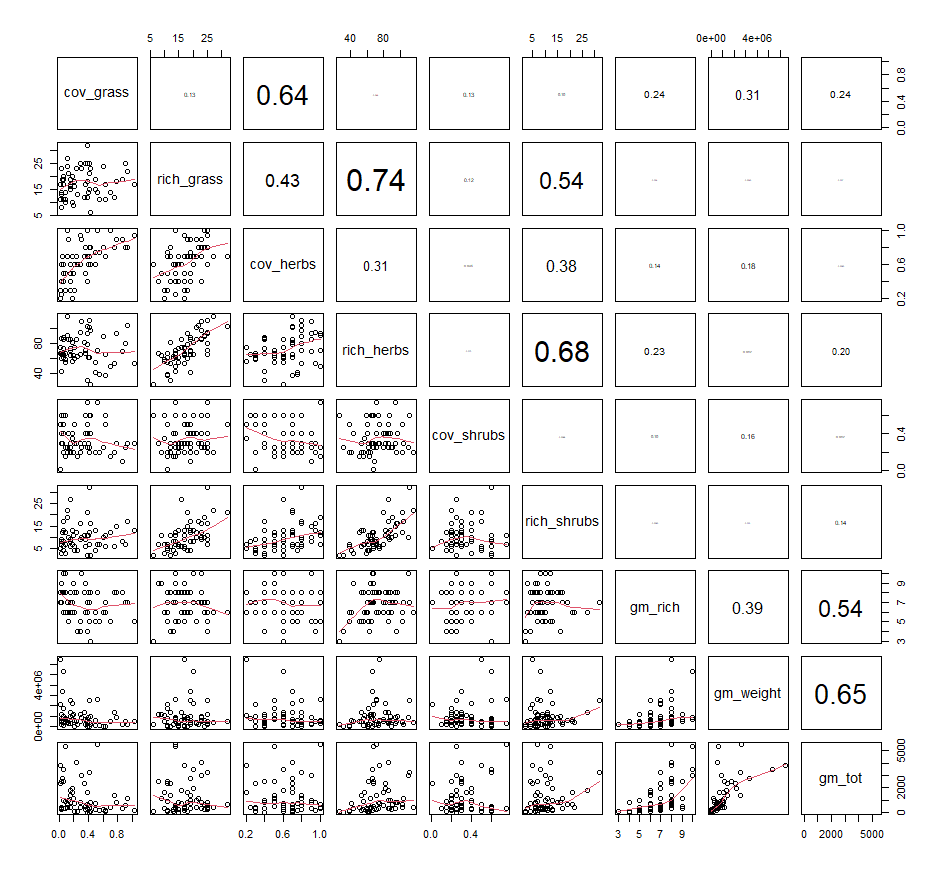


Correlations among selected variables including all responses and predictors. Correlation coefficients between pairs of variables are displayed in the upper right part of the panel with the font size corresponding to each correlation. In the lower left part of the panel the predicted correlations are shown by the red line depicting the relationship between the variables plotted using the Loess smoother. Variable abbreviations: **cov_grass** = cover of grasses, **rich_grass** = richness of grass species, **cov_herbs** = cover of herbs (including grasses), **rich_herbs** = richness of herb species (including grasses), **cov_shrubs** = cover of shrubs, **rich_shrubs** = richness of shrub species, **gm_tot** = number of grazer and mixed-feeder records, **gm_weight** = biomass of recorded grazers and mixed-feeders, **gm_rich** = number of grazers and mixed-feeders.

**Appendix S3**. Models testing the relationships between grass cover and species richness (responses) and grazer abundance, grazer species richness, habitat and bedrock (predictors). All mutual two-way interactions except herbivore abundance:herbivore species richness were included.

m1<-lme(sqrt(cov_grass) ~ scale(gm_tot)+scale(gm_rich)+habitat+bedrock+scale(gm_tot):habitat+scale(gm_tot):bedrock+scale(gm_rich):habitat+scale(gm_rich):bedrock+habitat:bedrock,random = ~ 1|triplet,data=envirn)

m2<-glmer(rich_grass ~ scale(gm_tot)+scale(gm_rich)+habitat+bedrock+scale(gm_tot):habitat+scale(gm_tot):bedrock+scale(gm_rich):habitat+scale(gm_rich):bedrock+habitat:bedrock+(1|triplet),family=poisson,data=envirn,control=glmerControl(optimizer="bobyqa",optCtrl=list(maxfun=2e5)))

**
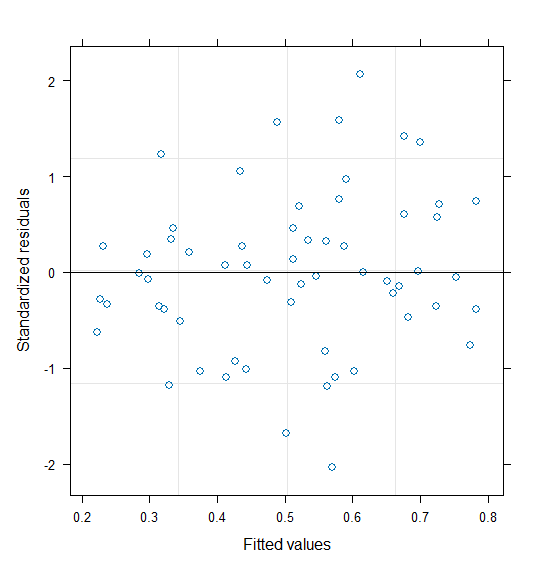
Appendix S4.** The Shapiro-Wilk tests did not reveal any significant deviations from normality, neither for the square-rooted response variable – grass species richness (W = 0.9669, p-value = 0.1027) nor the residuals (W = 0.98906, p-value = 0.8689). The residuals of square-rooted response do not show any signs of heteroskedasticity with respect to the fitted values, as shown in the diagnostic graph below.
